# Adverse health outcomes for prostate cancer patients treated with radiotherapy combined with androgen-deprivation therapy: A population-based, controlled study, from Norway

**DOI:** 10.2340/1651-226X.2025.42825

**Published:** 2025-08-25

**Authors:** Mona Nilsson, Anne Holck Storas, Tom Børge Johannesen, Ylva Maria Gjelsvik, Kirsti Aas, Sophie D. Fosså, Tor Å. Myklebust

**Affiliations:** a Cancer Registry of Norway, Norwegian Institute of Public Health, Oslo, Norway; b Institute of Clinical Medicine, Faculty of Medicine, University of Oslo, Oslo, Norway; c Department of Oncology, Oslo University Hospital, Oslo, Norway; d Department of Urology, Akershus University Hospital, Lørenskog, Norway; e Department of Research and Innovation, Møre and Romsdal Hospital Trust, Ålesund, Norway

**Keywords:** Prostate cancer, radiotherapy, androgen deprivation therapy, EPIC-26 domain summary scores, quality of life, late effects

## Abstract

**Background and purpose:**

The aim of this controlled cross-sectional, and population-based study was to evaluate adverse health outcomes (AHOs) 3 years after curative radiotherapy (RT) + androgen deprivation therapy (ADT). We also assessed Global Health/Quality of Life (QoL).

**Patients/material and methods:**

The Cancer Registry of Norway (CRN) provided data on prostate cancer (PCa) patients diagnosed in 2017–2019. All had been treated with RT+ ADT. All had completed EPIC-26 and EORTC QLQ-C30 about 3 years after RT start (n = 663). ADT duration was stratified: Short (< 9 months), intermediate (9–18 months) and long ADT (18–24 months). A group of controls were established from the general population (n = 1,817). Outcome measures were the urinary irritative/obstructive domain summary score (DSS), the bowel and sexual DSSs (EPIC-26) and QoL (EORTC QLQ-C30).

**Results:**

Compared to controls, patients had clinically important lower bowel, and sexual mean scores. Urinary irritative/obstructive DSS levels were similar. Overall, 43% (PCa patients) and 20% (controls) reported major sexual problems. In patients aged < 75 years, longer than short ADT duration significantly decreased sexual DSS. QoL was relatively unaffected. Low response rates, selection bias and a lack of pre-treatment data represent the studys´ limitations.

**Conclusion and Interpretation:**

Three years post-RT+ADT, PCa patients describe clinically important lower EPIC-26 bowel and sexual DSS compared to controls. Sexual domain levels decreased with increasing ADT duration, particularly in patients < 75 years. Our observations indicate worse AHOs than previously reported and should be considered during pre-treatment counselling of PCa patients.

## Introduction

High dose radiotherapy (RT) represents a curative treatment for prostate cancer (PCa) patients without distant metastases. RT is often combined with (neo-)adjuvant androgen deprivation therapy (ADT) of varying duration [1]. According to risk-classifications, short-term ADT (4–6 months) is recommended in intermediate-risk disease (Gleason 4+3), and long-term ADT (2–3 years) in high-risk PCa patients [1], with individual adaptations.

Multi-center studies of patient-reported outcomes (PROs) have shown that urinary symptoms, bowel dysfunction and sexual dysfunction are common post-RT long-term adverse health outcomes (AHOs) [2–8], accompanied by modest decrease in quality of life (QoL) [2, 7, 9, 10]. Discrepancies between described dysfunction and problem experience have been documented [2, 7, 9–13]. Previous research mainly represented PCa patients diagnosed before 2015 and included patients participating in formalized prospective studies. Improved RT-technology of recent years has not been accounted for in these older studies. Few RT studies have described self-reported AHOs in population-based real-word cohorts [10], and comparison with a normal population has rarely been done [11, 14–16]. Notably, the effect of varying duration of (neo-) adjuvant ADT use, is generally insufficiently described [17, 18].

There is thus a need for population-based knowledge of post-RT AHOs in patients treated after 2015, also describing the impact of on ADT duration.

Our controlled study therefore primarily evaluates the impact of modern curative RT combined with ADT on patient-reported pelvic AHOs 3 years after treatment start, with assessment of QoL as a secondary aim. For selected AHOs we quantify the discrepancy between dysfunction and problem experience.

## Patients and methods

### Patients

This study includes men diagnosed with PCa between 2017 and 2019, according to the Cancer Registry of Norway (CRN), who had undergone curative RT+ ADT, independent from their initial risk grouping, and completed a 3-year questionnaire assessing post-RT AHOs. The questionnaire was sent out approximately 3 years after the PCa diagnosis [19].

In all evaluable patients the local curative treatment had consisted of RT (defined as a 2 Gy equivalent total dose [EQD2] of at least 74 Gy), combined with (neo-) adjuvant ADT (Luteinizing hormone releasing hormone [LHRH] agonists or antagonists with or without oral anti-androgens). Since 2017 all Norwegian RT units have used Intensity-Modulated Radiation Therapy and Volumetric-Modulated Arc Therapy techniques. Data on type and duration of ADT were based on filed prescriptions in the Norwegian Prescribed Drug Registry. Accounting for individual variations during follow-up, three groups of ADT duration were identified in our data: short ADT (< 9 months), intermediate ADT (9–18 months), and long ADT (18–24 months). Patients were stratified according to age at questionnaire completion, < 75 versus ≥ 75 years. Risk groups were identified according to first-time registration in the CRN: ‘Intermediate’, ‘High risk, local’ and ‘High risk, locally advanced’, without consideration of the Gleason score.

The questionnaire included questions as to partnership, education (≥ college vs. < college) and experienced incidence of major comorbidity (cardiovascular disease, pulmonary disease, diabetes, others), and the result of the last PSA test. All patients stated that they had discontinued ADT.

### Controls

The CRN also invited men with no history of PCa, randomly drawn from the Norwegian population register, frequency matched on age and county of residence of the invited patients to complete the above questionnaires, except any PCa specific questions.

### Outcomes

In this study, the following three EPIC-26 domain summary scores (DSS) were calculated (https://medicine.umich.edu/): Urinary irritative/obstructive, bowel and sexual DSS [20]. The urinary incontinence DSS was excluded, due to documented small post-RT changes [2, 7]. Each DSS reflects the prevalence and severity of the domain’s AHOs, ranging from 0 (worst) to 100 (best). We highlighted the bowel and sexual problem items (item 7 and 12) [2, 9, 12], to separate the functional deficits from the man’s subjective reaction. Reporting moderate or big problems by item 7 or item 12, was defined as ‘Major problem’ related to bowel and sexual function [21]. Problem within the urinary irritative/obstructive DSS was not highlighted due to lack of a related problem item. We interpreted our intergroup differences by the nadir values of the published ranges of minimal clinically important differences (MCIDs) referred to as MCIDlow: Urinary irritative/obstructive > 5 points, Bowel function > 4 points and Sexual function > 10 points [22]. Global health/QoL was measured by combining the scores of item 29 and 30 of the EORTC QLQ-C30 [23]. Inter-group differences of QoL > 10 points were viewed as clinically important [24].

### Statistical methods

Descriptive statistics are presented by median and range for continuous variables and by absolute numbers and percentages for categorical variables. Multivariable linear regression models were estimated to adjust for differences between PCa patients and controls.

In addition to PCa patients versus controls, these models included the following covariates: Age (< 75 vs. ≥ 75), education (< college vs. ≥ college) and self-reported major comorbidity (Yes vs. No). From the estimated models we predicted the above outcome means across the covariate distribution, referred to as ‘adjusted means’. The correlation between a DSS and its problem item was expressed by Pearson’s R. Cronbach’s alpha was in all DSSs’ > 0.65 and was lowest in the urinary irritative/obstructive DSS (Supplementary Table 2). Statistical significance level: *p* < 0.05. Analyses were performed using STATA version 18.0 (StataCorp, College Station, TX, USA).

### Ethics

The study was approved by the Regional Ethical Committee of Health Region South-East in Norway (no. 2015/1294). Participants consented to inclusion in the study by returning the questionnaires.

## Results

**Figure 1:**
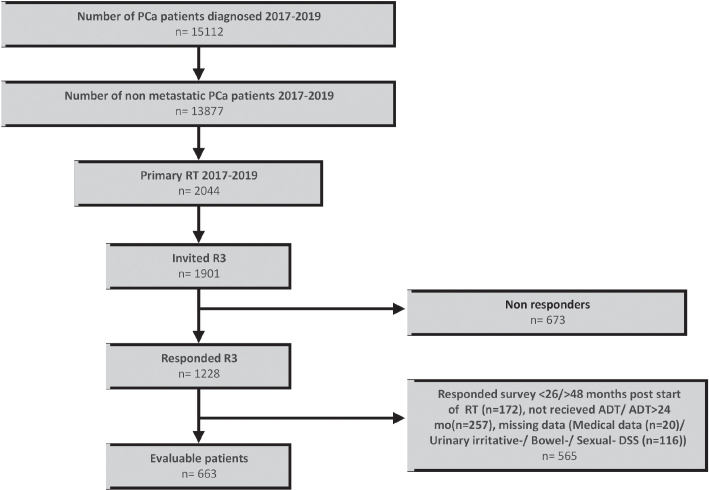
Flow chart showing patient inclusion. PCa: Prostate cancer, RT: Radical radiotherapy; R3: Three-years’ survey; ADT: androgen deprivation therapy.

In total, 663 (54%) of 1,901 invited PCa patients were evaluable (Figure 1). The mean response time was 2.9 years (SD; 0.2) after start of RT. In total, 2012 of 10,843 (19%) invited controls responded, resulting in 1,817 evaluable controls.

Patients were older than controls (median age 76 vs. 70 years), reported lower education and more comorbidity (Table 1). In total, 43% of the patients had received ADT for < 9 months, 24% for 9–18 months and 33% for 18–24 months. Hypofractionated RT was performed in 348 (53%) of the included patients (Table 1). Only small differences emerged between unadjusted and adjusted means (Table 2/Supplementary Table 1).

**Table 1:** Characteristics of survey participants.

Characteristic	PCa patients	Controls
	*n* = 663	*n* = 1,817
**Age at survey**		
All (median, IQR range)	76 (54, 89)	70 (45, 97)
< 75	267 (40%)	1,249 (69%)
≥ 75	396 (60%)	568 (31%)
Level of education, *n* (%)^*^		
≤ 12 years	282 (54)	624 (45)
> 12 years	236 (46)	762 (55)
Partnership, *n* (%)^*^		
Married/cohabitating	488 (81)	1,354 (80)
Single	111 (19)	341 (20)
**Self-reported comorbidity, *n* (%)**		
No	285 (43)	1,116 (61)
Yes	378 (57)	701 (39)
* Cardiovascular disease*	*124 (18)*	*271 (15)*
* Diabetes*	*84 (13)*	*191 (11)*
* Pulmonary disease*	*113 (17)*	*196 (11)*
Other^**^	*57 (9)*	*43 (2)*
**Risk group at diagnosis, *n* (%)**		
Intermediate^***^	174 (26)	
High risk, local	186 (28)	
High risk, locally advanced	303 (46)	
**Definite curative treatment, *n* (%)**		
EBRT	637 (96)	
RT	26 (4)	
**Androgen deprivation therapy (ADT), *n* (%)**		
All ADT	661 (100)	
* < 9 months*	*286 (43)*	
* 9–18 months*	*160 (24)*	
* 18–24 months*	*217 (33)*	
**Fractions**		
≤ 30	348 (53)	
> 30	315 (47)	

PCa: Prostate cancer; EBRT: External beam radiotherapy; BRT: Brachy radiation therapy.

* The numbers adds up to < 663 or < 1,817, due to missing data;

** HIV/AIDS, digestive, rheumatological, kidney or liver disease;

*** Gleason grouping unknown.

### Comparison between PCa patients and controls

#### Domain levels

**Figure 2:**
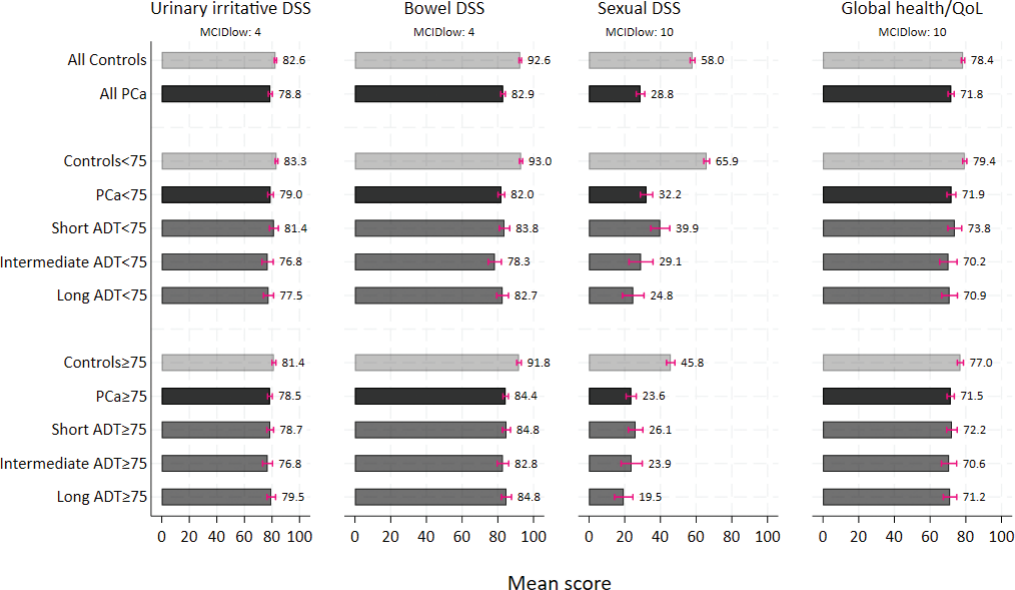
Predicted means from multivariable linear regression analysis displayed for all controls and PCa patients, by age groups and ADT duration, adjusted for education (< college vs. ≥ college) and self-reported comorbidity (yes vs. no). DSS: Domain summary scores; PCa: Prostate cancer; ADT: Androgen deprivation therapy; MCIDlow: Lowest minimal clinical important differences.

Table 2/Figure 2 documents the adjusted outcome means. The outcome scores for all PCa patients were lower (worse) than in controls, with clinically important differences for the bowel and the sexual domain (Table 2/Figure 2). No clinically important differences between PCa patients and controls emerged for the urinary irritative/obstructive DSS. For PCa patients Supplementary Table 2 documents clinical important differences for urinary irritative/obstructive DSS and global health between patients without self-reported comorbidity compared to those who described at least one comorbidity. PCa patients treated with hypofractionated RT (≤ 30 fractions) had a clinically significant better (higher) bowel DSS compared to patients treated with conventional RT (> 30 fractions) (85.3 vs. 81.0). No clinically significant differences emerged between hypofractionated RT and conventional RT for the other outcomes (Supplementary Table 2).

**Table 2:** Adjusted^*^ DSS means, problem means and prevalence, for controls and prostate cancer (PCa) patients </≥ 75 years, and for all controls and PCa patients.

Outcome	Age < 75 years		Age ≥ 75 years		All	
	Controls	PCa patients	Controls	PCa patients	Controls	PCa patients
**Urinary irritative/obstructive DSS (mean, CI)**	**83.3 (82.3, 84.3)**	**79.0 (76.9, 81.1)**	**81.4 (80.0, 82.8)**	**78.5 (76.8,80.2)**	**82.6 (81.7, 83.4)**	**78.8 (77.3, 80.2)**
*MCIDlow: 5*						
**Bowel DSS (mean, CI)**	**93.0 (92.1, 93.9)**	**82.0 (80.1, 83.9)**	**91.8 (90.6, 93.1)**	**84.4 (82.8, 85.9)**	**92.6 (91.8, 93.3)**	**82.9 (81.6, 84.2)**
*MCIDlow: 4*						
Related problem (Q7)^*^ (mean, CI)	**90.3 (88.9, 91.6)**	**77.4 (74.5, 80.2)**	**87.5 (85.6, 89.5)**	**79.2 (76.9, 81.6)**	**89.2 (88.1, 90.3)**	**78.1 (76.1, 80.1)**
*Related problem n (%)* ^**^	*40 (3)*	*34 (13)*	*25 (4)*	*35 (9)*	*65 (4)*	*69 (10)*
Correlation^***^	**0.87**	**0.87**	**0.87**	**0.85**	**0.87**	**0.86**
**Sexual DSS (mean, CI)**	**65.9 (64.3, 67.5)**	32.2 (28.8, 35.7)^*^	**45.8 (43.4, 48.1)**	**23.6 (20.8, 26.5)**	**58.0 (56.6, 59.3)**	**28.8 (26.5, 31.2)**
*MCIDlow: 10*						
Related problem (Q12)^*^ (mean, CI)	**69.1 (67.0, 71.1)**	**44.3 (39.8, 48.7)**	**62.3 (59.3, 65.4)**	**50.6 (46.9, 54.2)**	**66.4 (64.7, 68.2)**	**46.7 (43.7, 49.8)**
*Related problem n (%)* ^**^	*217 (17)*	*132 (50)*	*137 (24)*	*151 (38)*	*354 (20)*	*283 (43)*
Correlation^***^	**0.75**	**0.58**	**0.48**	**0.38**	**0.65**	**0.43**
**Global health/QoL (mean, CI)**	79.4 (78.2, 80.6)^*^	71.9 (69.4, 74.4)^*^	77.0 (75.2, 78.7)^*^	71.5 (69.5, 73.6)^*^	78.4 (77.5, 79.4)^*^	71.8 (70.0, 73.5)^*^

DSS: Domain summary scores; PCa: Prostata cancer; MCIDlow: Lowest minimal clinical important differences.

* Adjusted for age, education and self-reported comorbidity;

** Number of patients with major problems;

*** Correlation between DSS and related bother.

In PCa patients and controls, men aged < 75 years had significantly better (higher) scores of the sexual domain than older participants. Supplementary Table 3 documented higher (best) scores in the youngest men, aged 45–66. Means of the urinary irritative/obstructive DSS and the bowel DSS were similar in PCa patients below and above 75 years (Table 2/Figure 2).

#### Problem experience

The level of sexual problem experience (item 12) was worse in all PCa patients than in controls (66 vs. 46). Among all patients 43% described major sexual problems compared to 20% among controls (Table 2). Among PCa patients below 75 years the percentage of patients with sexual problems was 50%, compared to 38% in the PCa patients ≥ 75 years (Table 2). Supplementary Table 3 documented least problems in the youngest age group (45–66 years). The correlation between sexual DSS and related problems was moderate (R: 0.43) in patients and 0.65 in controls (Table 2). The lowest correlation was observed in the oldest patients (R: 0.38). In contrast, the problem experience within the bowel domain and the high item-domain correlation (*R* > 0.80) was not related to the man’s age.

### Impact of ADT duration (DSS; problem experience)

Among patients we found lower (worse) sexual means with increasing ADT duration. Only in the youngest PCa patients, the differences reached clinical importance (Figure 2). Among the young PCa patients the percentage of patients with major sexual problems was 54 and 52% in the intermediate and long ADT subgroups, respectively, compared to 39 and 35% in the PCa patients ≥ 75 years (Table 3). Despite some variations in the bowel domain, no clinical important differences within the ADT subgroups emerged (Figure 2).

**Table 3:** Adjusted^*^ problem means, and prevalence of problem for ADT duration subgroups, </≥ 75 years.

Outcome	Age < 75 years				Age ≥ 75 years			
	Short ADT	Intermediate ADT	Long ADT	All	Short ADT	Intermediate ADT	Long ADT	All
**Overall bowel problem (Q7) (mean, CI)**	**77.7 (72.2, 83.2)**	**73.6 (66.6, 80.6)**	**78.1 (71.8, 84.3)**	**76.7 (73.2, 80.3)**	**79.0 (74.8, 83.2)**	**78.1 (72.0, 84.2)**	**78.3 (73.0, 83.7)**	**78.6 (75.7, 81.5)**
***Related problem n (%)***	*15 (14)*	*9 (13)*	*10 (11)*	*34 (13)*	*14 (8)*	*12 (13)*	*9 (7)*	*35 (9)*
Correlation^**^	**0.87**	**0.92**	**0.85**	**0.87**	**0.81**	**0.90**	**0.86**	**0.85**
**Overall sexual problem (Q12) (mean, CI)**	**45.2 (37.6, 52.8)**	**42.2 (32.7, 51.8)**	**42.3 (33.7, 50.9)**	**43.5 (38.6, 48.4)**	**48.8 (43.1, 54.6)**	**47.5 (39.1, 55.9)**	**52.6 (45.2, 60.0)**	**49.7 (45.7, 53.6)**
***Related problem n (%)***	*49 (45)*	*37 (54)*	*46 (52)*	*132 (50)*	*70 (40)*	*36 (39)*	*45 (35)*	*151 (38)*
Correlation^**^	**0.69**	**0.59**	**0.44**	**0.58**	**0.38**	**0.48**	**0.37**	**0.38**

ADT: Androgen deprivation therapy.

* Adjusted for age, education and self-reported comorbidity;

** Correlation between DSS and related problem.

### Global health/QoL

We found no clinical important difference in global health/QoL between PCa patients and controls (Table 2/Figure 2). The difference was larger for the youngest participants, though the difference did not reach clinical importance.

## Discussion

Three years after curative prostatic RT, combined with ADT and using EPIC-26, we documented lower levels of EPIC-26 bowel and sexual domain in PCa patients than in controls. Differences between PCa patients and controls highly exceeded MCIDlow (Bowel DSS: 9.7, Sexual DSS: 29.2). The sexual DSS decreased with rising age and increasing duration of ADT. The bowel DSS levels were in patients and controls highly reflected by the men’s problem experience (*r* > 0.80), with less correlation regarding the sexual DSS (*r*: 0.43). In total, 10% of PCa patients, and 4% of controls reported major bowel problems, the corresponding prevalence of sexual problems being 43% in PCa patients and 20% in controls. The urinary irritative/obstructive DSS scores were similar in PCa patients and controls. Despite these differences related to bowel and sexual function in patients versus controls, differences of QoL between PCa patients and controls were not clinically significant. Our findings as to bowel and sexual domain are worse than previously reported results.

### Methodological aspects

The discussion of our results must separate the effect of pelvic RT from the impact of the duration of androgen effect, both objectives being the principal aims in this study.

Bowel dysfunction is primarily related to RT performance. Whether new techniques reduce patient-reported adverse effects compared to older methods, is still to be documented. In this study over half of the PCa patients had high risk cancer, often treated with long-lasting ADT, a target dose of 78 Gy and target fields, which frequently include the pelvic lymph nodes. Such RT increases the risk of radiation-induced atherosclerosis of the pelvic vessels, which is viewed as the main reason for post-RT sexual dysfunction [25–27]. Large target fields increase scattered testicular irradiation, which delays post-ADT testosterone recovery. Except for bowel DSS, where PCa patients with hypofractionated RT had a clinically significant better (higher) score compared to conventionally RT, no clinically significant differences emerged between hypofractionated RT and conventional RT in this study. This finding is in line with the literature, describing small differences between the two RT approaches, and overall concluding that hypofractionated RT offer biologically effective dose in a shorter time, without increased toxic effects [28]. Better bowel DSS in hypofractionated patients was somewhat unexpected. PCa patients treated with conventionally RT were slightly older and had more health problems (higher Eastern Cooperative Oncology Group (ECOG) performance status) compared to the patients treated with hypofractionated RT, this might have influenced the results.

### Comparison between PCa patients and controls

#### Domain levels

We found clinically important differences in PCa patients and controls related to the bowel and sexual domain 3 years after RT, with the greatest differences observed for the sexual domain. A few older, but no recent studies covering modern RT technology, have compared post-RT+ADT self-reported AHOs with the corresponding AHOs reported by age-similar controls [11, 14, 15, 29]. These studies demonstrated differences between PCa patients and controls, comparable to this study.

The small difference within the urinary irritative/obstructive DSS, is in line with previous findings [16, 28]. One explanation for this overall similarity between patients and controls may be that the 3-year post-RT interval is too short for the development of symptom-inducing fibrosis. Also, low item-test correlations in 2 of 5 items related to this domain affect the domains’ overall validity.

Compared to results from older studies (Table 4), and one contemporary study in 1-year PCa survivors [9], our overall means in PCa patients are worse, though heterogeneity of socio-demographic variables, tumor characteristics and treatment years, makes direct comparisons problematic. Comparable to previous findings [28] we observed some age-dependency (< 75 vs. ≥ 75 years). Of note, our national cohort of PCa patients reflects ‘real life practice’, whereas most of the previous studies include patients from prospective, multicenter studies. The high age and increased comorbidity of our PCa patients are likely to have contributed to these differences.

**Table 4:** Published observations for EPIC-26 urinary irritative/obstructive subscale, bowel- and sexual DSS, and sexual bother, 2–3 years after curative radiotherapy combined with ADT in PCa patients.

First author (ref.nr)	Treatment period	Mean/median age	Observation time/#Pca	Urinary irritative/obstructive	Bowel DSS	Sexual DSS
Sanda et al. (2008) (PROSTQA)	2003–2006	69^3^	2 years, *n* = 292	88^*1^	90^*1^	30^*1^
Donovan et al. (2023) (ProtecT)^**^	2001–2009	62^3^	3 years, *n* = 473	93^1^	91^1^	43^1^
Barocas et al. (2017) (CEASAR)^***^	2011–2012	68^3^	3 years, *n* = 598	86^1^	90^1^	39^1^
Caumont et al. (2020)	2007–2017	71^3^	3 years, *n* = 71	–	85^*2^	18^*2^
Current study	2017–2019	75	3 years, *n* = 663	79	83	29

DSS: Domain summary scores; ADT: Androgen deprivation therapy; PCa: Prostate cancer;

* Numbers were extracted from a figure;

** Randomized controlled trial;

*** Population based;

1 Unadjusted values;

2 Among others adjusted for age, race, baseline HRQoL, comorbidity,

3 Age at study start.

#### Problem experience

Compared to previous studies, our PCa patients reported more problems than controls, especially within the sexual domain [11, 14]. Lower (worse) sexual problem scores in the younger patients and controls (< 75 years) compared to ≥ 75 years, implies that sexual dysfunction is of greater relevance for these men.

Comparable to previous findings [2, 7–12] sexual dysfunction in PCa patients was only limitedly reflected by their problem experience, except for the youngest patients. The generally lower correlations within the sexual domain may indicate higher acceptance of sexual AHOs 3 years after curative cancer treatment in aging PCa patients. In contrast, the correlations regarding bowel problems were high in PCa patients and controls.

#### Impact of ADT duration

As anticipated, the duration of ADT had limited impact on urinary irritative/obstructive and bowel DSS, whereas great variations emerged for the sexual domain according to ADT duration, modified by age. As in previous studies we observed the highest (best) levels of sexual DSS in PCa patients aged < 75 years after short ADT.

This observation suggests better recovery ability in these patients, together with the longer ADT-free period than in the remaining men [17, 18]. Despite differences in the definitions of short- and long-term ADT treatment, our findings of the sexual domain are comparable to previous studies.

#### Global health/QoL

No clinically important difference between PCa patients and controls was observed, in line with previous published reports [15, 16]. Also, the QoL differences stratified for ADT duration were generally small. This may indicate that the overall QoL is to a small degree affected by the selected AHOs.

## Strengths and limitations

There are several limitations of this study. Firstly, low response rates and the risk of selection bias, particularly among controls, must be acknowledged. However, in our controls we observed similar levels of AHOs and dysfunctions as a study on the male population in Northern Ireland [30] (Supplementary Table 6). PCa patients planned for curative treatment should have a life expectancy of at last 10 years [1], which potentially results in a selection bias by identification of healthier patients than age-similar controls.

Supplementary Table 5 indicates a possible selection by age, as the participating PCa patients were older than the non-participants. Secondly, this study does not have sufficient information about the size or configuration of the radiation target fields, the use of sexual aids, or most importantly, about the patients’ pretreatment sexual function [10, 31]. The population-based, real-world design, including contemporary RT patients grouped according to ADT duration comparing the results with age-similar controls, and the adjustment for confounding factors, are the main strengths of this study.

Finally, initial risk grouping, as documented in the CRN, had probably not in all PCa patients reflected the tumor risk at RT start, explaining the use of ADT in all patients.

## Conclusion

Three years after RT combined with ADT, lower (worse) Epic 26-scores are documented in the bowel and sexual domains compared with controls, the differences being clinically important. The Sexual DSS decreased (worsened) with extended ADT duration, but the probem experience was similar across ADT subgroups. We document greater prevalence of post-treatment bowel and sexual AHOs than previously reported. Our findings should be considered during pre-treatment counselling of PCa patients who are candidates for RT+ADT.

## Supplementary Material



## Data sharing

Data are not available to other researchers. Registry or institutional database of patients routinely collected data.
