# Supplementary material for: Adverse health outcomes for prostate cancer patients treated with radiotherapy combined with androgen-deprivation therapy: A population-based, controlled study, from Norway
Source: Acta Oncol. 2025 Aug 25;64:42825. doi: 10.2340/1651-226X.2025.42825 (PMC12398109; doi:10.2340/1651-226X.2025.42825)
Supplement: Supplementary file 1 [file AO-64-42825-s1.pdf]

Supplementary material has been published as submitted. It has not been copyedited, or typeset by Acta Oncologica

Suppl. Table 1. Unadjusted DSS means, for controls and PCa </≥75 years, and for all controls and PCa patients.

	Age <75 years		Age≥75 years		All	
	Controls *Mean (CI)*	PCa patients *Mean (CI)*	Controls *Mean (CI)*	PCa patients *Mean (CI)*	Controls *Mean (CI)*	PCa patients *Mean (CI)*
**Urinary obstructive/ irritative DSS**	84.1 (81.3,85.0)	78.7 (76.5,80.8)	81.4 (80.1,82.7)	78.5 (77.0,80.1)	83.3 (82.6,84.0)	78.6 (77.3,79.9)
**Bowel DSS**	93.0 (92.3,93.6)	81.5 (79.2,83.8)	92.0 (90.1,93.0)	83.4 (81.7,85.1)	92.7 (92.1,93.2)	82.7 (81.3,84.0)
**Related problem**	90.2 (89.2,91.2)	76.7 (73.4,79.9)	87.3 (85.6,89.1)	78.5 (76.0,81.0)	89.3 (88.4,90.2)	77.8 (76.4,79.1)
**Sexual DSS**	67.4 (65.9,68.8)	30.6 (27.4,33.8)	45.0 (42.7,47.2)	22.9 (20.9,24.9)	60.4 (59.1,61.7)	26.0 (24.2,27.7)
**Related problem**	69.7 (68.0,71.4)	41.8 (37.8,45.9)	62.8 (60.1,65.5)	50.4 (46.8,54.0)	67.6 (66.1,69.0)	46.9 (44.2,49.7)
**Global health/ QoL**	79.8 (78.8,80.8)	69.0 (66.4,71.6)	75.9 (74.2,77.6)	70.8 (68.8,72.7)	78.6 (77.7,79.5)	70.1 (68.2,71.6)

Suppl. Table 2. Adjusted DSS stratified by comorbidity and hypofractionated RT.

	Urinary obstructive/ irritative DSS	Bowel DSS	Sexual DSS	Global health
**Controls**				
No comorbidity	84.8 (83.8,85.9)	94.6 (93.6,95.5)	62.8 (61.0,64.5)	83.5 (82.2,84.7)
One or more comorbidity	79.7 (78.4,81.0)	90.1 (88.9,91.2)	52.2 (50.1,54.4)	72.1 (70.5,73.6)
**PCa patients**				
No comorbidity	80.9 (78.8,82,9)	84.3 (82.5,86.2)	32.8 (29.4,36.2)	77.0 (74.6,79.4)
One or more comorbidity	76.4 (74.7,78.2)	81.9 (80.3,83.5)	26.6 (23.7,29.6)	65.7 (63.6,67.8)
Hypofractionated RT (≤30 fractions)	79.0 (75.8,82.1)	85.3 (82.2,88.4)	28.6 (24.6,32.7)	70.0 (66.4,73.6)
Hypofractionated RT (>30 fractions)	77.1 (74.8,79.4)	81.1 (78.8,83.3)	23.1 (20.2,26.0)	67.5 (64.9,70.1)

Suppl. Table 3. Adjusted* DSS means, problem means and prevalence, for all Controls and all PCa patients, and divided into quartiles.

	45-66 years		67-73 years		74-76 years		77-97 years		All	
	Controls	PCa patients	Controls	PCa patients	Controls	PCa patients	Controls	PCa patients	Controls	PCa patients
**Urinary obstructive/ irritative subscale (mean, CI)**	**84.6 (83.1,86.0)**	**80.8 (75.4,86.3)**	**82.4 (81.0,83.9)**	**78.2 (75.2,80.8)**	**82.1 (80.1,84.0)**	**79.7 (77.0,82.5)**	**80.9 (79.1,84.0)**	**78.1 (76.1,80.1)**	**82.6 (81.7,83.4)**	**78.8 (77.3,80.2)**
MCIDlow: 5										
**Bowel DSS (mean, CI)**	**92.7 (91.4,94.0)**	**81.8 (77.0,86.7)**	**93.0 (91.7,94.3)**	**83.1 (80.8,85.4)**	**92.9 (91.7,94.3)**	**82.2 (79.7,84.6)**	**91.8 (90.2,93.4)**	**84.5 (82.7,86.3)**	**92.6 (91.8,93.3)**	**82.9 (81.6,84.2)**
MCIDlow: 4										
**Related problem (Q7)* (mean, CI)**	**90.7 (88.7,92,7)**	**78.6 (71.2,86.1)**	**89.6 (87.6,91.6)**	**78.1 (74.6,81.7)**	**88.7 (86.1,91.4)**	**77.7 (73.9,81.4)**	**87.7 (85.2,90.2)**	**79.1 (76.4,81.9)**	**89.2 (88.1,90.3)**	**78.1 (76.1,80.1)**
*Related problem n(%)***	18 (3%)	4 (9%)	19 (3%)	24 (13%)	11 (4%)	14 (9%)	17 (5%)	27 (9%)	*65 (4%)*	*69 (10%)*
**Correlation*****	**0.87**	**0.88**	**0.87**	**0.88**	**0.88**	**0.88**	**0.86**	**0.84**	**0.87**	**0.86**
**Sexual DSS (mean, CI)**	**72.5 (70.1,74.8)**	**45.2 (36.5,53.9)**	**62.2 (59.9,64.6)**	**29.5 (25.3,33.6)**	**51.6 (48.5,54.8)**	**29.9 (25.5,34.3)**	**42.3 (39.4,45.2)**	**21.6 (18.4,24.8)**	**58.0 (56.6,59.3)**	**28.8 (26.5,31.2)**
MCIDlow: 10										
**Related problem (Q12)* (mean, CI)**	**73.2 (70.1,76.3)**	**54.6 (33.0,56.2)**	**66.2 (63.1,69.2)**	**45.1 (39.7,50.5)**	**61.9 (57.8,66.0)**	**45.4 (39.6,51.2)**	**62.9 (59.1,66.6)**	**51.6 (47.4,55.9)**	**66.4 (64.7,68.2)**	**46.7 (43.7,49.8)**
*Related problem n(%)***	95 (16%)	19 (44%)	103 (18%)	90 (51%)	66 (22%)	70 (47%)	90 (25%)	104 (36%)	*354 (20%)*	*283 (43%)*
**Correlation*****	**0.82**	**0.75**	**0.71**	**0.53**	**0.67**	**0.55**	**0.36**	**0.34**	**0.65**	**0.43**
**Global health/ QoL (mean, CI)**	**78.7 (77.0,80.5)**	**70.7 (64.2,77.3)**	**79.8 (78.1,81.5)**	**71.8 (68.8,74.9)**	**79.2 (76.9,81.5)**	**72.7 (69.4,76.0)**	**76.2 (74.0,78.3)**	**71.2 (68.8,73.6)**	**78.4 (77.5,79.4)***	**71.8 (70.0,73.5)***

*Adjusted for education and self-reported comorbidity; DSS, Domain Summary Scores; PCa, Prostata Cancer; MCIDlow, Lowest Minimal Clinical Important Differences; **Number of patients with major problems; ***Correlation between DSS and related bother.

Suppl. Table 4. Item- test correlation.

	Item-test correlation*	Alpha**
**Urinary irritative/ obstructive DSS**		
Problem with pain or burning on urination(Q4b)	0.2831	0.7153
Problem with hematuria(Q4c)	0.1300	0.7293
Problem with weak urine stream/ incomplete emptying(Q4d)	0.8202	0.5493
Problem with urine frequence(Q4e)	0.8641	0.5167
Overall urinary problem(Q5)	0.8459	0.5035
**Test scale**		**0.6852**
**Bowel DSS**		
Problem with urgency(Q6a)	0.8589	0.7954
Problem with increased frequency(Q6b)	0.8593	0.7912
Problem with losing control(Q6c)	0.7290	0.8248
Problem with bloody stools(Q6d)	0.4552	0.8685
Problem with abdominal/pelvic/rectal pain(Q6e)	0.6663	0.8391
Overall bowel problem(Q7)	0.8723	0.7872
**Test scale**		**0.8468**
**Sexual DSS**		
Ability to have an erection(Q8a)	0.9022	0.9251
Ability to reach an orgasm(Q8b)	0.9326	0.9195
Quality of erections(Firmness)(Q9)	0.9532	0.9152
Frequency of erections(Q10)	0.6259	0.9645
Overall sexual function(Q11)	0.9413	0.9180
Overall sexual problem(Q12)	0.9137	0.9222
**Test scale**		**0.9397**

*Item–test correlation, The correlation between a scored item and the total test score; **Cronbach' alpha without the specified single item.

Suppl. Table 5. Unadjusted patient characteristics among all PCa patients diagnosed 2017-2019 and undergoing RT, survey responders, and non-invited/ invited non-participants.

	All patients registered with RT in CRN*	Survey participants	Non- participants
	n= 3877[a]	n= 663[a]	n= 3214[a]
**Age**			
All *(median, IQR range)*	71 (37,89)	76 (54,89)	
<75	2794 (72%)	267 (40%)	2527 (79%)
≥75	1083(28%)	396 (60%)	687 (21%)
**ECOG**			
0	2558 (79%)	516 (81%)	2034 (79%)
≥1	672 (21%)	120 (19%)	550 (21%)
**Risk group**			
Low/intermediate	935 (28%)	174 (26%)	761 (28%)
High risk, local	907 (27%)	186 (28%)	721 (27%)
High risk, locally advanced	1517 (45%)	303 (46%)	1214 (45%)

PCa, Prostate Cancer; RT, radiotherapy; *non invited and invited/ Not invited due to logistical errors; [a]The numbers adds up to <3877, <663 or <3214, due to missing data

Suppl. Table 6. Published observations on EPIC-26 items in prostate cancer free men.

	Donnelly et al. (2018), %<br>Age ≥60 years	Current study, %<br>Median age 70 (45,97)
Pain or burning on urination	1.7	0.4
Bleeding with urination	0.3	0.3
Incomplete emptying	9.1	13.0
Need to urinate frequently	16.6	15.2
Problem with urinary function	9.3	9.0
Urgency to have a bowel movement	6.7	4.6
Increased frequency of bowel movements	5.0	3.0
Losing control of bowel movements	2.2	1.0
Bloody stools	0.6	0.4
Abdominal, pelvic, rectal, back passage pain	3.1	2.0
Problem with bowel habit	6.5	3.6
Very poor or no ability to have an erection	27.8	34.1
Very poor or no ability to reach orgasm	26.2	27.6
Never have an erection when wanted	33.6	23.8
Very poor ability to function sexually	33.0	32.9
Problem with sexual function	32.8	19.5
